# The extent of metalloproteinase-mediated LAG3 cleavage limits the efficacy of PD1 blockade

**DOI:** 10.1186/2051-1426-3-S2-P216

**Published:** 2015-11-04

**Authors:** Lawrence P Andrews, Andrea L Szymczak-Workman, Creg J Workman, Dario AA Vignali

**Affiliations:** 1 University of Pittsburgh, Pittsburgh, PA, USA

## 

Inhibitory receptors control immune responses preventing exacerbated T cell activation and the onset of autoimmunity; however, they also limit antitumor immunity. Enhanced co-expression of PD1 and LAG3 phenotypically mark functionally exhausted tumor-specific T cells, with dual PD1/LAG3 blockade synergistically limiting tumor growth in murine models. Like PD1, LAG3 expression is induced on activated T cells to negatively regulate T cell activation and proliferation and LAG3 is also required for maximal regulatory T (T_reg_) cell function. However, LAG3 expression and function is itself regulated by cell surface cleavage of the transmembrane domain connecting peptide by ADAM10 and ADAM17 metalloproteinase-disintegrins. This releases soluble LAG3, of which no biological function has been found to date. To investigate the impact of LAG3 cleavage on T cells within tumors, a non-cleavable LAG3 mouse (LAG3.NC) was generated in which exons 7 and 8 of *Lag3*, including the connecting peptide, is deleted in Cre-expressing cells. These exons are replaced and modified so that the connecting peptide is absent preventing LAG3 cleavage. LAG3.NC CD4^Cre^ mice (with non-cleavable LAG3 expressed on all CD8^+^ and CD4^+^ T cells, including T_regs_) and LAG3.NC E8I^Cre^ mice (restricted to CD8^+^ T cells only) exhibit enhanced expression of LAG3 on the respective T cell subsets in B16-F10 or MC38 tumors, co-expressing with PD1. Despite increased LAG3 expression, no difference in B16-F10 or MC38 tumor growth was observed in either LAG3.NC CD4^Cre^ or LAG3.NC E8I^Cre^ mice compared with wild-type littermates. Upon therapeutic administration of anti-PD1 antibody (clone G4), MC38 tumor-bearing wild-type mice show significant tumor regression and 40% become tumor-free resulting in long-term survival. LAG3.NC CD4^Cre^ mice were resistant to anti-PD1 therapy and succumb to tumor growth. However, anti-PD1 mediated tumor regression and long-term survival in LAG3.NC E8I^Cre^ mice. Analysis of re-stimulated CD8^+^ TILs isolated from LAG3.NC CD4^Cre^ mice did not show enhanced IFN-gamma and TNF-alpha production following anti-PD1 therapy, which was observed with LAG3.NC E8I^Cre^ mice or wild-type littermates. Moreover, reduced proliferation was observed for all T cell subsets in LAG3.NC CD4^Cre^ mice compared with LAG3.NC E8I^Cre^ and wild-type littermates following anti-PD1 treatment. As LAG3.NC CD4^Cre^, but not LAG3.NC E8I^Cre^ mice, are resistant to the favorable antitumor immune effects induced by anti-PD1, this suggests that enhanced LAG3 expression on CD4^+^ T cells or T_regs_ may act as a barrier to effective anti-PD1 immunotherapy. LAG3.NC mice crossed with Cre that restricts non-cleavable LAG3 to T_regs_ (Foxp3^yfpiCre^) or CD4^+^ T cells (ThPOK^Cre^) are currently under analysis.

